# Paeonol Attenuated Inflammatory Response of Endothelial Cells via Stimulating Monocytes-Derived Exosomal MicroRNA-223

**DOI:** 10.3389/fphar.2018.01105

**Published:** 2018-11-20

**Authors:** Yarong Liu, Chao Li, Hongfei Wu, Xianmei Xie, Ying Sun, Min Dai

**Affiliations:** ^1^School of Pharmacy, Anhui University of Chinese Medicine, Hefei, China; ^2^Key Laboratory of Xin’an Medicine, Ministry of Education, Hefei, China

**Keywords:** paeonol, atherosclerosis, lipopolysaccharide, exosomes, microRNA-223, STAT3, monocytes, endothelial cells

## Abstract

**Introduction:** Paeonol, an active compound isolated from the radix of *Cortex Moutan*, has been shown to have anti-atherosclerosis effects by regulating blood cells’ function and protecting vascular cells injury. Besides, emerging evidences has proven that exosomes might play a pivotal role in intercellular communication by transmiting proteins and microRNAs from cell to cell. However, the relationship between monocytes-derived exosomal microRNA-223 and vascular inflammation injury along with paeonol’ effects are still not clear.

**Objective:** Our study aimed to explain whether paeonol’s protective effect on inflammatory response is related to the regulation of exosomal microRNA-223 in the VECs.

**Methods:** ApoE^−/−^ mice were fed with high fat diet to replicate the AS model. HE staining and immunohistochemistry was used to detect inflammatory response of aorta. The expression of IL-1β and IL-6 were detected by ELISA. Western blot was used to detect the expression of STAT3, pSTAT3, ICAM-1 and VCAM-1. qRT-PCR was used to detect miR-223 expression. Exosomes were extracted from THP-1 cells by differential centrifugation and observed by transmission electron microscope. Observation of exosomes uptake into HUVECs was realized by laser microscopy. miR-223 target gene was detected by double luciferase gene report test.

**Results:** *In vivo* experiments confirmed that paeonol restricted atherosclerosis development and increased miR-223 expression, inhibited STAT3 pathway in ApoE^−/−^ mice. *In vitro*, miR-223 showed robust presence in THP-1 cells and undetectable in HUVECs. And we had observed that miR-223 could be internalized from THP-1 cells into HUVECs taking exosomes as a carrier. Paeonol obviously increased miR-223 expression in co-cultured HUVECs and exosomes in concentration dependent manner, compared to LPS group. In addition, paeonol relieved inflammatory secretion, adhesion and STAT3 expression in HUVECs, which could be inverted after miR-223 inhibitor transfection into THP-1 cells.

**Conclusion:** Paeonol could increase the expression of miR-223 in THP-1 derived exosomes and in HUVECs after uptake of exosomes, whereas decrease the expression of STAT3, p-STAT3 in HUVECs. Ultimately paeonol decreased the expression of IL-1β, IL-6, ICAM-1, VCAM-1 in HUVECs and alleviated adhesion of THP-1 cells to HUVECs.

## Introduction

Atherosclerosis (AS) is a chronic inflammatory disease involving multiple factors and multiple cells. Dysfunction and inflammation of vascular endothelial cells (VECs) are the driving forces in the initiation and development of AS (Peter, 2002; Briasoulis et al., 2012). Lipopolysaccharide (LPS), as one of the risk factors of AS, can activate the innate immunity of monocyte/macrophage (M/MΦ), resulting in VECs damage. Recently there have been considerable studies focusing on exosomes, which are released by M/MΦ and regulate the receptor cells function (Moore and Tabas, 2011; Orekhov et al., 2016).

Exosomes are nano-sized biological membrane-enclosed vesicles that contain a cell-specific cargo of proteins, DNA, mRNA and microRNA (miRNA) that are released and taken up by most cell types, thereby inducing expression and functional changes via transfer of cargos between cells (Valadi et al., 2007; Sluijter et al., 2014). In proinflammatory conditions such as AS and other cardiovascular diseases, exosomes derived from VECs, vascular smooth muscle cells, macrophages, and other circulating immune cells mainly possess proinflammatory properties.

miRNAs are single-stranded, non-coding small RNAs, which are involved in almost all phases of AS, from lesion initiation, through progression, to ultimately clinical complications of this disease (Santovito et al., 2012). Here, we look at the miRNA(miR)-223’s emerging role in vascular damage. In this respect, it is interesting that miR-223 was found to be upregulated in AS patients (Meng et al., 2012). miR-223 levels were found to be correlated with classical biomarkers of AS, such as cholesterol, urea and calcium levels (Taïbi et al., 2014). As the report shown, in the atherosclerotic ApoE^−/−^ mice models, an obvious decrease of miR-223 was observed in aortic atherosclerotic lesions and in LPS activated macrophages, its expression was decreased (Wang et al., 2015). Furthermore, miR-223 was recently described as a regulator of cholesterol uptake (Meyer et al., 2013). Recent studies have revealed that miR-223 is nearly exclusively expressed in hematopoietic cells at bone marrow and in bone marrow-derived blood cells, mainly in blood platelets and leukocytes. Although vascular cells should have no endogenous miR-223 expression, a significant amount of miR-223 has been identified in normal vascular walls, because of the transfer carrier, exosomes. Exosomes released by monocytes have been proven to contain a high amount of miR-223 (Haneklaus et al., 2013). Exosomes loaded with abundant miR-223 from monocytes can be shuttled into the endothelial cells in a biologically anti-inflammation effect (Li et al., 2014; Tabet et al., 2014). miR-223 may inhibit the development of AS by regulating its downstream signal transducers and activators of transcription 3 (STAT3) and other inflammatory target genes. Therefore, as a regulatory factor in the upstream of STAT3, miR-223 may be a new target for the treatment of AS (Yin et al., 2013; Qin et al., 2015).

Paeonol (2′-hydroxy-4′-methoxyacetophenone, C_9_H_10_O_3_) is one of the main active compound isolated from the radix of *Cortex Moutan*, the chemical structure as shown in Figure 2. An emerging evidences exhibit the anti-inflammatory effects of paeonol. Our previous studies showed that paeonol protected against endothelial injury stimulated by LPS or oxidized low density lipoprotein (ox-LDL) via decreasing the inflammatory secretion of tumor necrosis factor-α (TNF-α), interleukin (IL-1), IL-6 (Chen et al., 2014; Liu et al., 2014). Besides, we have confirmed that paeonol could exert anti-AS effects by inhibiting monocytes adhesion to VECs (Wang et al., 2012; Yuan et al., 2016). However, investigations regarding the material exchanges between monocytes and VECs after adhesion have never been reported. In our study, we attempted to explore the role of human acute monocytic leukemia cell line (THP-1) derived exosomal miR-223 in inflammatory response of VECs and clarify the underlying mechanisms of paeonol’s protective effect on vascular inflammation *in vivo* and *in vitro*.

## Materials and Methods

### Ethics Statement

All surgical and experimental procedures were approved by the Ethics Review Committee for Animal Experimentation of the Institute of Clinical Pharmacology at Anhui Medical University.

### Animal Model

ApoE^−/−^ mice on C57BL/6 background, weighing from 17 to 27 g, were used to build the atherosclerosis animal model and purchased from the Department of Laboratory Animal Science, Vital River, Beijing, China. The 6-week-old male mice were kept under standard laboratory conditions [temperature 22–25°C and relative humidity 55–65%) with 12:12 dark/light and fed a high-cholesterol diet (HCD, containing 21% fat and 0.15% cholesterol] until atherosclerotic lesions were obviously formed on arteries. Subsequently, atherosclerotic animals were randomly put into 2 groups (*n* = 8 mice/group). One group was oral administrated with 10 mg/kg of paeonol in contained 5% CMC-Na solution daily for total 6 weeks, the other group with CMC-Na solution as model group. C57BL/6 mice were fed normal diet throughout the experiment as control group. Mice were ultimately killed by exsanguination before blood samples and arteries were collected.

### Chemicals and Reagents

Paeonol (99% purity) was obtained from Baicao Plants Biotech Co, Ltd (Anhui, China). LPS, GW4869 and Heparin were all purchased from Sigma–Aldrich (United States). The cell culture materials and fetal bovine serum (FBS) were obtained from GIBCO BRL (Gaithersburg, United States). The BCA Protein Assay kit was obtained from Shanghai Haoran Bio Technologies Co, Ltd (China). HiPerFect transfection reagent, MiRNeasy Mini Kit and MiScript PCR Starter Kit were obtained from Qiagen (Germany). miR-223 inhibitor and mimic were purchased from zoonbio Biotechnology Co. Ltd (Nanjing, China). The dual-luciferase reporter system (Cat. E1910) was purchased from Promega Company (United States). Antibodies against STAT3, p-STAT3 and VCAM-1 were obtained from Cell Signaling Technology Co. Ltd (United States). Antibodies against ICAM-1, CD9, ALIX, TSG101 and Calnexin were purchased from Abcam Co, Ltd. (United States).

### Hematoxylin and Eosin (HE) Staining

The aortas of ApoE^−/−^ mice were excised and fixed before embedded by paraffin. Then the paraffin-embedded aortas were cut into 4 μm thick sections and dewaxed. Subsequently, the sections were stained with hematoxylin for 6 min and eosin for another 1 min. After dehydration, resinene was used to seal the sections to transparency. The analysis was performed by inverted microscope (×20) and quantitated with the Image J software according to artery lumen area.

### Co-culture System of THP-1 Cells and HUVECs

We co-cultured THP-1 cells with HUVECs in a Transwell system with a 0.4 μm porous membrane (Corning, Corning, NY, United States). The HUVECs were planted in the lower wells and grown for an appropriate period of time. The THP-1 were then planted in the upper wells. Thus the intercellular communication was realized between THP-1 cells and HUVECs without direct connection.

### CCK8 Assay

THP-1 cells were planted in 96-well plates (5 × 10^4^ cells/mL) and pretreated by paeonol (7.5, 15, 30, 60, 120 μmol/L) for (6, 12, 24, 36, 48 h) respectively, then 1 μg/mL LPS for another 24 h. The cells were incubated for 4 h with fresh medium containing 10 μL CCK-8 reagent per well. Then the absorbance at 450 nm was measured by an enzyme-linked immunoadsorbent assay microplate reader (Synergy 2, Bio-TEK).

### Exosomes Isolation and Labeling

Exosomes were isolated by differential centrifugation. THP-1 cells were centrifuged at 500 *g* for 5 min at room temperature before the supernatant was removed and centrifuged at 1500 *g* for 15 min to pellet the cellular debris. The resultant supernatant was aspirated then centrifuged at 100,000 *g* for 90 min at 4°C to pellet exosomes. Purified exosomes were washed with PBS and labeled with the green fluorescent linker PKH67 (Sigma).

### Nanoparticle Tracking Analysis

Exosomes size distribution was determined with a Nanosight NS500 nanoparticle analyzer (Malvern Instruments) equipped with a 405 nm laser. Analysis was performed with NTA 3.1 software.

### Adherence Assay

HUVECs (1 × 10^5^ cells/well) were seeded in 12-well plates until 80∼95% confluent. THP-1 cells were labeled with 5 μM Vybrant DiO (V22885 Invitrogen^TM^) for 30 min at 37°C. Labeled THP-1 cells were then co-cultured with HUVECs for 2 h. Finally, co-cultured cells were washed with PBS containing 1% bovine serum albumin (BSA). Images were captured using a fluorescence microscope (Leica, Germany).

### Enzyme-Linked Immunosorbent Assay (ELISA)

The expression levels of IL-1β and IL-6 in HUVECs were analyzed by ELISA according to the manufacturer’s protocol. The cells supernatant was collected for detection and the solution absorbance at 450 nm was determined by a microplate reader. A standard curve was generated according to the standard solution, which was used for linear regression analysis by Microcal Origin 8.0 software.

### Laser Scanning Confocal Microscopy

THP-1 derived exosomes were labeled with PKH67 as per manufacturer’s instructions, then the labeled exosomes were washed twice with PBS and concentrated. HUVECs were seeded into 6-well plates and labeled exosomes were added. For confocal microscopy, samples were fixed with 4% methanol-free paraformaldehyde for 10 min, permeabilized in 0.1% Triton-X 100 in PBS for 3 min. After fixation and permeabilization, cells were washed thrice with PBS, blocked in 1% bovine serum albumin in PBS for 10 min, and stained with a solution containing 0.5 unit/μL Alexa Fluor 488-conjugated phalloidin for 20 min at room temperature. MicroRNA or exosomes were visualized through DAPI staining.

### Western Blotting

Protein concentrations were determined by BCA Protein Assay kit. The proteins were seperated by 10% SDS-polyacrylamide gel electrophoresis and transferred onto nitrocellulose membranes, which were blocked for 3 h at room temperature or overnight at 4°C with 5% blocking solution. After incubation with antibodies, the membranes were washed and incubated with secondary antibodies of mouse/rabbit conjugated to horseradfish peroxidase. The immunoreactive proteins were visualized by the enhanced chemiluminescence.

### RNA Extraction and qRT-PCR Assay

Total RNA was extracted using QIAzol Lysis Reagent according to the manufacturer’s protocol for miR-223 analyses. For detection of gene expression, qRT-PCR was performed using Quantitect SYBR Green PCR Kits. Relative expression was evaluated by comparative CT method and normalized to the expression of U6 small RNA. Changes in gene expression were determined using the 2^−ΔΔCt^ method.

### Dual-Luciferase Reporter Assay

The sequence for miR-223 can be predicted from Pubmed gene database, the matched sites are shown below. Luciferase-reporter plasmids contained wild type or mutant 3′-UTR segments of STAT3 were constructed to verify the putative binding site of miR-223. The wild type or mutant reporter plasmid was cotransfected into HUVECs along with miR-223 mimic. After 24 h, luciferase activity was measured using the dual luciferase assay system. The firefly luciferase activity of each sample was normalized to Renilla reniformis luciferase activity.

### Statistical Analysis

Results were presented as mean ± SEM and analyzed by SPSS 21.0 (United States). The Student’s *t*-test was used for statistical comparisons between two groups, while one-way ANOVA for multiple groups. A difference was considered statistically significant when *P* < 0.05.

## Results

### Paeonol Restricted Atherosclerosis Development and Increased the miR-223 Expression of Aorta in ApoE^−/−^ Mice

**FIGURE 1:**
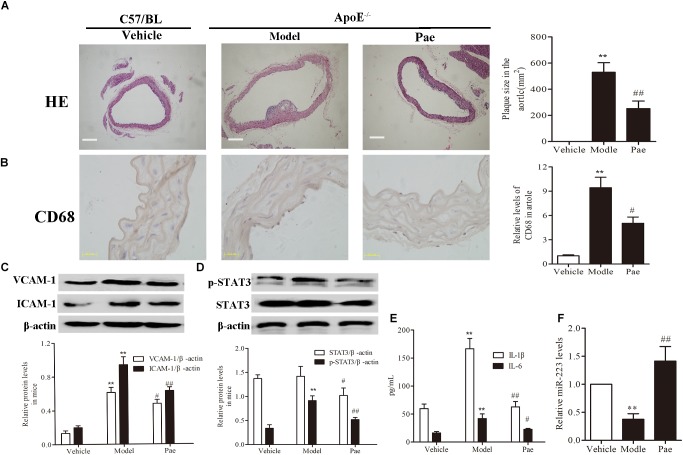
Paeonol restricted atherosclerosis development and increased the miR-223 expression of aorta in ApoE^−/−^ Mice. The dosage of paeonol was 10 mg/kg body weight. **(A)** H&E staining and quantification of plaque areas. Bar = 20 μm. **(B)** Adhesion and infiltration of mononuclear macrophages in the aorta represented by CD68 expression level using immunocytochemistry. Bar = 20 μm. **(C)** VCAM-1 and ICAM-1 proteins expression in the aorta by Western blot. **(D)** STAT3 and p-STAT3 proteins expression in the aorta by Western blot. **(E)** IL-1β and IL-6 levels in the serum of ApoE^−/−^ mice. **(F)** miR-223 expression of aorta. Data are the mean ± SEM, n = 3. ^∗∗^*P* < 0.01 vs. Vehicle group, ^#^*P* < 0.05, ^##^*P* < 0.01vs. Model group. Paeonol was given 10 mg/kg body weight.

First, to evaluate the effect of paeonol on plaque formation and inflammatory reaction, our group detected a range of indicators of AS in ApoE^−/−^ mice. The dosage of paeonol was 10 mg/kg body weight based on our previous study (Wu et al., 2017). The results indicated that paeonol could significantly reduce AS plaque formation (*P <* 0.01) (Figure 1A). Result of CD68 protein expression revealed that paeonol could remarkably inhibit the adhesion and infiltration of aortic macrophages in ApoE^−/−^ mice (Figure 1B). Meanwhile, paeonol obviously suppressed the release levels of IL-1β, IL-6, VCAM-1, ICAM-1 in ApoE^−/−^ mice from the result of ELISA assay and Western Blot assay (Figures 1C,E). Furthermore, we explored paeonol’s effects on miR-223 and STAT3 in aorta of ApoE^−/−^ mice by the means of immunocytochemical method and western blot. Compared to model group, paeonol increased the miR-223 level (Figure 1F). Conversely, paeonol significantly inhibited the expression of STAT3 and pSTAT3 (Figure 1D). Taken together, our results suggested that paeonol treatment restricted AS lesion development and inflammatory reaction in ApoE^−/−^ mice. However, whether paeonol’s therapeutic effect on AS was related to the augment of miR-223 and inhibition of STAT3 pathway deserved further investigation.

### Paeonol Decreased Inflammatory Response of HUVECs in the Co-culture System With THP-1 Cells

**FIGURE 2:**
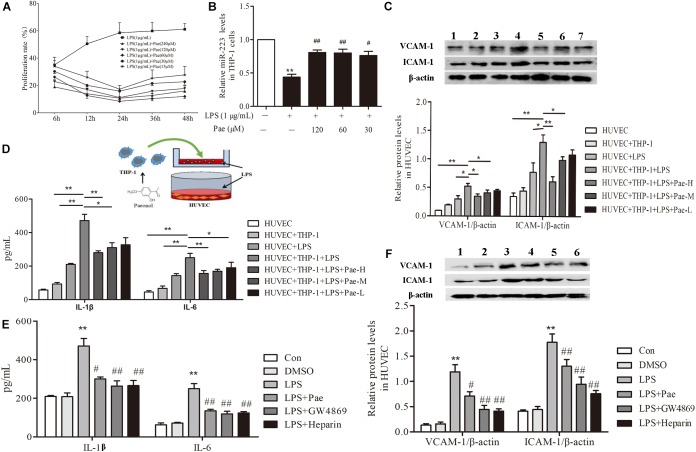
Paeonol decreased inflammatory response of HUVECs in the co-culture system with THP-1 Cells. **(A)** THP-1 cells were treated with the indicated concentration of paeonol (15–240 μM) for different period of time (6, 12, 24, 36, 48 h) in the presence of LPS (1 μg/mL). The optimal dosage of paeonol was detected by CCK8 assay with THP-1 cells proliferation as an index. **(B)** The expression of miR-223 in LPS-stimulated THP-1 cells. The data were expressed as mean ± SEM, *n* = 6. ^∗∗^*P* < 0.01 vs. Control group, ^#^*P* < 0.05, ^##^*P* < 0.01 vs. LPS group. **(C)** (1) HUVEC group. (2) HUVEC + THP-1 group. (3) HUVEC + LPS group. (4) HUVEC + THP-1 + LPS group. (5) HUVEC + THP-1 + LPS + Pae-H group. (6) HUVEC + THP-1 + LPS + Pae-M group. (7) HUVEC + THP-1 + LPS + Pae-L group. THP-1 cells were pretreated by paeonol (30, 60, 120 μM) for 24 h and LPS (1 μg/mL) for another 24 h. Then HUVECs were co-cultured with the THP-1 cells. The VCAM-1 and ICAM-1 levels in co-cultured HUVECs. **(D)** The IL-1β and IL-6 levels in co-cultured HUVECs. Data are the mean ± SEM, *n* = 3. ^∗∗^*P* < 0.01 vs. HUVEC group, ^ΔΔ^*P* < 0.01 vs. HUVEC + LPS group, ^#^*P* < 0.05, *^##^P* < 0.01 vs. LPS group. **(E)** The effects of GW4869 (10 μM) and Heparin (10 μg/mL) on IL-1β and IL-6 levels in co-cultured HUVECs. The concentration of paeonol was 60 μM. **(F)** (1) Control group. (2) DMSO group. (3) LPS group. (4) LPS + Pae group. (5) LPS + GW4869 group. (6) LPS + Heparin group. The effects of GW4869 (10 μM, 8 h) and Heparin (10 μg/mL, 24 h) on VCAM-1 and ICAM-1 levels in co-cultured HUVECs. ^∗∗^*P* < 0.01 vs. HUVEC group, ^##^*P* < 0.01, ^#^*P* < 0.05 vs. LPS group.

In order to determine the best concentration and time of paeonol, we pretreated THP-1 cells with paeonol at diverse concentration for various lengths of time. According to previous study, 1 μg/mL LPS stimulated THP-1 cells for 24 h acting as model group. Our results indicated that an optimal condition was established when the cells were pretreated with paeonol (30, 60, 120 μM) for 24 h (Figure 2A). Consistently, miR-223 expression results also showed that paeonol (30, 60, 120 μM) could significantly increase the expression of miR-223 in LPS-stimulated THP-1 cells at 24 h (Figure 2B), which was employed in the following experiments.

To evaluate crosstalk between THP-1 cells and HUVECs, we established a co-culture system and detected paeonol’s effects on Inflammatory secretion in HUVECs using ELISA assay and Western Blot assay. Compared to monoculture HUVECs, LPS stimulation led to higher release levels of IL-1β, IL-6, VCAM-1, ICAM-1 in co-cultured HUVECs. Remarkably, paeonol could reduce those inflammatory factors’ level more significantly in co-cultured HUVECs than monoculture HUVECs (Figures 2C,D), suggesting the co-culture system established successfully. Accordingly, we speculated that exosomes secreted by THP-1 cells were involved in the inflammatory response of HUVECs. 10 μM GW4869 was incubated with THP-1 cells for 8 h to block the exosomes secretion, while 10 μg/mL Heparin was incubated with HUVECs for 24 h to block the exosomes uptake. Treatment of GW4869 and Heparin could decrease inflammatory response of co-cultured HUVECs compared to LPS-stimulated group, consistent with paeonol (*P <* 0.01) (Figures 2E,F). These data indicated that paeonol decreased inflammatory response of HUVECs potentially through THP-1 derived exosomes.

### Paeonol Enhanced miR-223 Expression of HUVECs in the Co-culture System With THP-1 Cells

**FIGURE 3:**
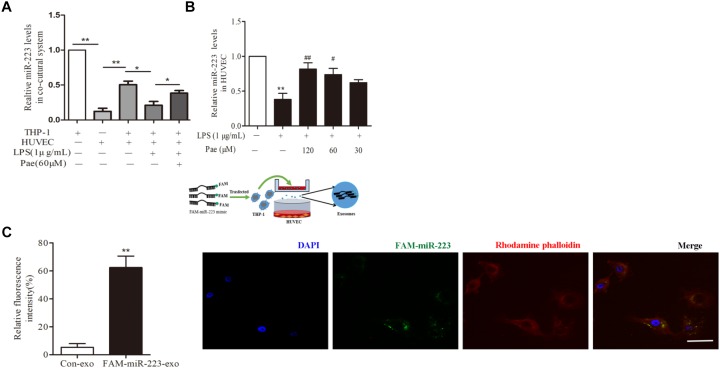
Paeonol enhanced miR-223 expression of HUVECs in the co-culture system with THP-1 cells. The data were expressed as mean ± SEM, *n* = 3. **(A)** miR-223 expression of HUVECs in the co-culture system. **(B)** The effects of paeonol on miR-223 expression of HUVECs in the co-culture system. ^∗∗^*P* < 0.01 vs. Control group, ^#^*P* < 0.05, ^##^*P* < 0.01 vs. LPS group. **(C)** Laser scanning confocal microscopy was used to observe the uptake of FAM-miR-223-exo by HUVEC. Bar = 10 μm. ^∗∗^*P* < 0.01 vs. Control group.

To observe the role of miR-223 in the crosstalk between HUVECs and THP-1 cells, HUVECs were co-cultured with THP-1 cells which transfected with FAM-miR-223 mimic. Then laser scanning confocal microscopy was used to observe the uptake of FAM-miR-223-exo by HUVECs. Statistic results were shown in Figure 3C, miR-223 could be internalized from THP-1 cells into HUVECs (*P <* 0.01). Subsequently, we performed a qRT-PCR to detect miR-223 expression in HUVECs. Remarkably, miR-223 showed robust presence in THP-1 cells, but was undetectable in HUVECs. After co-cultured with THP-1 cells, miR-223 expression of HUVECs was much higher than monoculture HUVECs (*P <* 0.01) (Figure 3A). Additionally, paeonol had obviously reversed the suppressed miR-223 expression caused by LPS in co-cultured HUVECs (*P <* 0.05) (Figure 3B).

### The Biological Morphology of the Exosomes Remain Unchanged Under LPS or Paeonol Incubation

**FIGURE 4:**
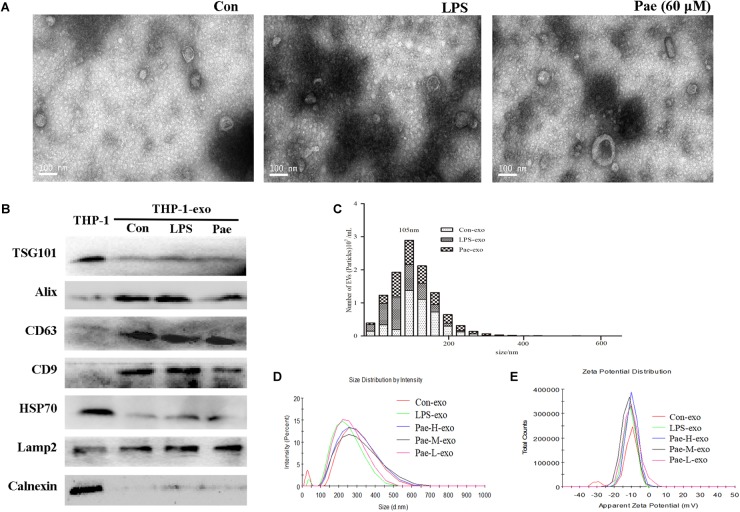
The biological morphology of the exosomes remain unchanged under LPS or paeonol incubation. The data were expressed as mean ± SEM, *n* = 6. **(A)** The morphology of exosomes was imaged using electron microscopy and the diameter of particles. Bar = 100 nm. **(B)** Exosomal markers TSG101, Alix, CD63, CD9, HSP70, Lamp2 and endoplasmic reticulum marker Calnexin were detected in cell and exosomes lysates by western blot. **(C)** Particle concentration distribution of exosomes by NTA. **(D,E)** Size distribution and surface ζ potential of exosomes secreted from cells by DLS.

In order to verify the effect of paeonol on the biological morphology of the exosomes, we characterized the exosomes by a variety of methods. For isolating exosomes, THP-1 cells were treated accordingly. The exosomes were isolated from the conditioned culture medium by differential centrifugation. Using electron microscopy, we observed the cup-shaped vesicles of exosomes with a typical diameter of 30–100 nm (Figure 4A), showing that LPS and paeonol has on effect on the morphology of exosomes. The expressions of the exosomes markers, including CD9, LAMP2, CD63, Alix, TSG101, HSP70 were then confirmed by Western blot. Results declared that marker proteins expression in exosomes were significantly higher than that of the THP-1 cells, which is counter to the expression of negative control protein Calnexin. In addition, LPS and paeonol groups had on significant difference on those proteins compared to control group (Figure 4B). To further investigate the size distribution profile of the THP-1-derived exosomes, we performed size analysis using the nanoparticle tracer analyzer and dynamic light scattering, revealing no significant differences between the groups with a peak size at about 105 nm and surface zeta potential value about −10 (Figures 4C–E). According to this, we speculated that exosomes isolated from THP-1 cells were stable under LPS or paeonol incubation.

### Paeonol Alleviated Inflammatory Response of HUVECs by Increasing THP-1 Derived Exosomal miR-223 in HUVECs

**FIGURE 5:**
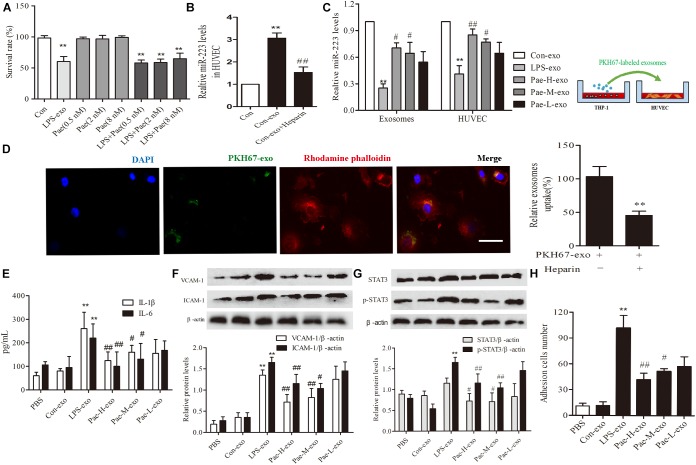
Paeonol alleviated inflammatory response of HUVECs by increasing THP-1 derived exosomal miR-223 in HUVECs. The data were expressed as mean ± SEM, *n* = 3. **(A)** The effect of a trace of paeonol (0.5, 2, 8 nM) on the activity of HUVECs and LPS-exo stimulated HUVECs. **(B)** Heparin (10 μg/mL) was pre-incubated with HUVECs for 24 h. HUVECs were co-cultured with THP-1 derived exosomes. qPCR was used to detect miR-223 levels in HUVECs. ^∗∗^*P* < 0.01 vs. Control group. ^##^*P* < 0.01 vs. Con-exo group. **(C)** THP-1 cells were pretreated by paeonol (30, 60, 120 μM) for 24 h and LPS (1 μg/mL) for another 24 h. The exosomes were extracted from THP-1cells, then HUVECs were co-cultured with the exosomes. miR-223 levels were detected in exosomes and HUVECs.^∗∗^*P* < 0.01 vs. Con-exo group, ^#^*P* < 0.05, ^##^*P* < 0.01 vs. LPS-exo group. **(D)** HUVECs were incubated with PKH-67 labeled exosomes. Confocal laser scanning was used to observe the uptake of HUVEC exosomes. Bar = 10 μm. **(E)** IL-1β, IL-6 levels of HUVECs co-cultured with THP-1 derived exosomes. **(F)** VCAM-1, ICAM-1 levels of HUVECs co-cultured with THP-1 derived exosomes. **(G)** STAT3 and p-STAT3 proteins levels of HUVECs co-cultured with THP-1 derived exosomes. **(H)** HUVECs were co-cultured with the exosomes for 24 h, then were added the THP-1 cells before the adhesion THP-1 cells number in HUVECs were detected. ^∗∗^*P* < 0.01 vs. Con-exo group, ^#^*P* < 0.05, ^##^*P* < 0.01 vs. LPS-exo group.

In order to exclude the interference of the paeonol remained in THP-1 derived exosomes on downstream experiments, we detected and calculated the quantification of paeonol in exosomes by HPLC (Table 1). Survival rate of HUVECs was detected by CCK8 assay, a trace of paeonol (0.5, 2, 8 nM) in exosomes had no effect on the activity of HUVECs and no therapeutic effects on LPS-exo stimulated HUVECs (Figure 5A).

**Table 1:** Paeonol quantification in exosomes (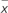 ± *s*, *n* = 6).

Pae treatment (μM)	Pae in exosomes (ng/μg)
240	13.5361 ± 0.0962
120	5.5032 ± 0.0296
60	2.4934 ± 0.0584
30	0.9671 ± 0.0055
15	0.6713 ± 0.0093

To further examine the endocytic pathways involved in HUVECs and THP-1 derived exosomes, we labeled exosomes with PKH-67 and incubated with HUVECs. Laser scanning confocal microscopy was used to observe the uptake of PKH-67 labeled exosomes and detect the uptake rate of exosomes in HUVECs. Figures illustrated that HUVECs could uptake exosomes from THP-1 cells (Figure 5D). Subsequently, we conducted qPCR assay, the miR-223 expression of HUVECs co-cultured with exosomes was increased obviously compared to control group, while exosomes uptake inhibitor Heparin could reverse the tendency (*P <* 0.01) (Figure 5B). Then THP-1 cells were stimulated by LPS and paeonol (30, 60, 120 μM) before THP-1 derived exosomes being extracted respectively, HUVECs were co-cultured with those exosomes. Paeonol could enhance miR-223 expression in exosomes dose-dependently compared to LPS-exo group, consistent with the miR-223 expression in HUVECs (Figure 5C). Thus, we demonstrated that paeonol enhanced miR-223 expression in HUVECs by means of THP-1 derived exosomes.

To further validate the effects of THP-1 derived exosomes on HUVECs inflammatory response and STATA3 pathway, we conducted following experiments in HUVECs-exosomes co-culture system. HUVECs were co-cultured with the exosomes for 24 h, then the secretion of inflammatory factors and adhesion were detected. LPS-exo could trigger the secretion of inflammatory factors in HUVECs and adhesion between THP-1 cells and HUVECs, conversely paeonol-exo could relieve the inflammatory response dose-dependently (Figures 5E,F,H). Similar result was found in STAT3 and pSTAT3 expression, LPS-exo could increase STAT3 and pSTAT3 expression in HUVECs and paeonol-exo reversed it in a dose-dependent manner (Figure 5G).

### STAT3 Was a Target Gene of miR-223 in HUVECs

**FIGURE 6:**
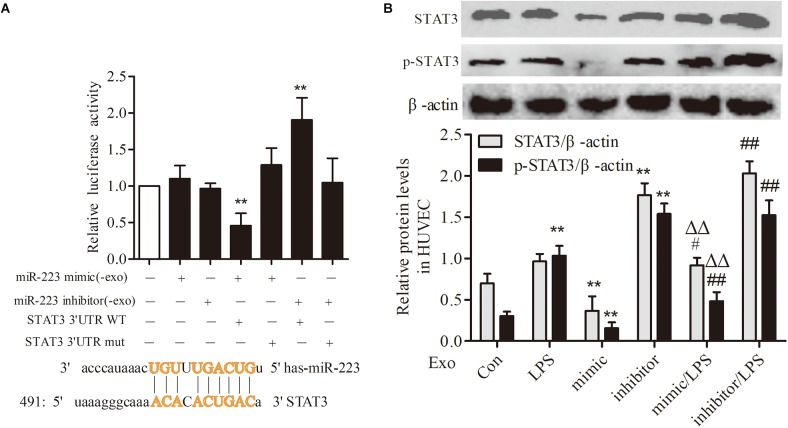
STAT3 was a target gene of miR-223 in HUVECs. The data were expressed as mean ± SEM, *n* = 3. **(A)** The wild type or mutant reporter plasmid was cotransfected into HUVECs with miR-223 mimic. ^∗∗^*P* < 0.01 vs. mimic group. **(B)** STAT3 and p-STAT3 expression in HUVECs. ^∗∗^*P* < 0.01 vs. Con group, ^#^*P* < 0.05, ^##^*P* < 0.01 vs. LPS group, ^ΔΔ^*P* < 0.01 vs. mimic group.

To investigate the association between THP-1 derived exosomal miR-223 and STAT3 pathway in HUVECs, we constructed luciferase-reporter plasmids containing wild type or mutant 3′-UTR segments of STAT3. The test results showed that the STAT3 was a downstream target gene of miR-223 in HUVECs (Figure 6A). After transfection of miR-223 mimic into THP-1 cells, the expression of STAT3 and pSTAT3 of HUVECs were reduced dramatically, while miR-223 inhibitor increased the tendency greatly (Figure 6B). These data revealed a negative correlation between miR-223 and STAT3 pathway.

### Paeonol Inhibited STAT3 Pathway by Increasing THP-1 Derived Exosomal miR-223 in HUVECs

**FIGURE 7:**
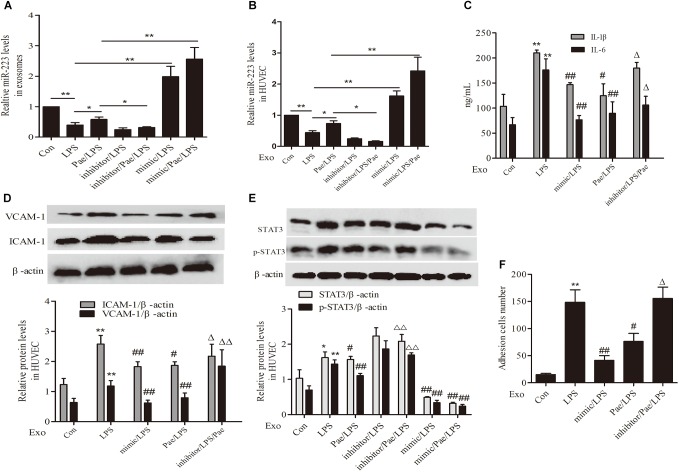
Paeonol inhibited STAT3 pathway by increasing THP-1 derived exosomal miR-223 in HUVECs. The data were expressed as mean ± SEM, *n* = 3. We performed transfection of miR-223 mimic or inhibitor to THP-1 cells, then THP-1 cells were treated by paeonol (60 μM) for 24 h and LPS (1 μg/mL) for another 24 h. Exosomes were extracted from THP-1 cells to establish the co-culture system of exosomes-HUVECs. **(A,B)** miR-223 levels in exosomes and co-cultured HUVECs. ^∗^*P* < 0.05, ^∗∗^*P* < 0.01. **(C)** IL-1β, IL-6 levels of co-cultured HUVECs. **(D)** VCAM-1, ICAM-1 levels of co-cultured HUVECs. **(E)** STAT3 and p-STAT3 proteins levels of co-cultured HUVECs. **(F)** Adhesion cells number of co-cultured HUVECs. ^∗∗^*P* < 0.01 vs. Con group, ^#^*P* < 0.05, ^##^*P* < 0.01 vs. LPS group, ^ΔΔ^*P* < 0.01 vs. Pae/LPS group.

To further validate the mechanism of paeonol’s effect on inflammatory response of HUVECs, We performed transfection of miR-223 mimic or inhibitor to THP-1 cells, then THP-1 cells were treated by paeonol (60 μM) for 24 h and LPS (1 μg/mL) for another 24 h. Exosomes were extracted from THP-1 cells to establish the co-culture system of exosomes-HUVECs. Results including the negative control of transfection of miR-223 mimic or inhibitor can be seen in Supplementary Figure 1. From the results (Figures 7A,B,E), paeonol could enhance the miR-223 expression and inhibit STAT3 pathway in both exosomes and HUVECs, which could be inverted after miR-223 inhibitor transfection into THP-1 cells. Afterward, we investigated the inflammatory response of HUVECs. In comparison with control group, LPS could increase the level of IL-1β, IL-6, VCAM-1, ICAM-1 and adhesion in HUVECs, which were restrained by miR-223 mimic transfection into THP-1 cells. Paeonol could alleviate inflammatory response of HUVECs and miR-223 inhibitor transfection into THP-1 cells could aggravate inflammatory response of HUVECs (Figures 7C,D,F). These data provided evidences that paeonol alleviated STAT3-mediated inflammatory response of HUVECs via increasing THP-1 derived exosomal miR-223 in HUVECs. Supplementary Presentation 1 shows the original Western blots images of the full text.

## Discussion

The major findings in this study were as follows. First, paeonol augmented miR-223 expression and inhibited STAT3 pathway in aorta, which contributing to alleviation of AS lesion development and inflammatory reaction of ApoE^−/−^ mice *in vivo*. Second, paeonol reduced inflammation secretion significantly in HUVECs co-cultured with THP-1 cells or THP-1 derived exosomes. Third, miR-223 could be internalized from THP-1 cells (where miR-223 was abundant) into HUVECs (where miR-223 was nearly undetectable) by means of exosomes, while paeonol could enhance the miR-223 expression of both exosomes and co-cultured HUVECs. Lastly, paeonol inhibited STAT3 pathway in HUVECs via upregulating THP-1 derived exosomal miR-223. Our research firstly proposed a new perspective about the effect of paeonol on VECs inflammation by regulating THP-1 derived exosomal microRNA.

In our study, we sought to uncover mechanisms by which paeonol could suppress vascular inflammation in progress of AS. To this end, we detected the secretion function of inflammatory molecules (IL-1β, IL-6) and adhesion molecule (VCAM-1, ICAM-1), as well as adhesion function, to reflect the extent of inflammatory response. Relentless research efforts have confirmed endothelial injury can lead to AS and various forms of cardiovascular risk factors can disturb the homeostasis in VECs. This vascular impairment leads to a dysfunctional endothelium, marked by the increased inflammatory response (Park et al., 2013; Khalyfa et al., 2016; Mongkoldhumrongkul et al., 2016; Ong et al., 2018). Increased secretion of inflammatory cytokines, such as IL-1β and IL-6, occurs due to VECs injury, which can regulate the expression and differentiation of multiple genes and accelerate the process of AS (Alsaffar et al., 2018). Meanwhile, ICAM-1 and VCAM-1 promote inflammation through mediating monocytes adhesion to the endothelial cells and eventually turning into foam cells (Lawson and Wolf, 2009; Fenyo and Gafencu, 2013). In our study, we proved that paeonol could obviously decrease the expression level of IL-1β, IL-6 and VCAM-1, ICAM-1, as well as adhesion function *in vivo* and *in vitro*.

Exosomes are lipid bilayer vesicles that range from 40 to 100 nm in diameter, which play an important role in autoimmune and inflammatory diseases. They serve as a communication tool for extracellular signaling over far distances and as a cargo for safe delivery of various biomolecules, including various types of proteins, RNAs, microRNA, etc (Yuan et al., 2017). Exosomes can be used not only as a marker of disease diagnosis, drug targeting transport carrier, is also expected to become a therapeutic target for diseases (Milane et al., 2015; Zhang et al., 2015; Suchorska and Lach, 2016). Several studies showed that miR-223 could transfer from monocytes to vascular cells by means of exosomes and play a pivotal role in normal physiological processes and pathological processes of AS (Cheng et al., 2017; Li et al., 2018; Liu et al., 2018). The miR-223 overexpression significantly attenuated macrophage foam cell formation, lipid accumulation and pro-inflammatory cytokine production (Bao et al., 2014). Our results revealed that treatment of GW4869 (exosomes secretion inhibitor) and Heparin (exosomes uptake inhibitor) could decrease inflammatory response in co-cultured HUVECs. Additionally, we observed that exosomes released from THP-1 cells transmit miR-223 into HUVECs. After miR-223 mimic transferred into THP-1 cells, the inflammatory response in co-cultured HUVECs was decreased and vice versa. Taken together, THP-1 derived exosomes transfer miR-223 into HUVECs, exerting anti-inflammation effect. Furthermore, our results clarified that paeonol could up-regulate miR-223 expression in THP-1 derived exosomes, therefore enhance miR-223 expression in HUVECs.

The downstream inflammatory target genes of miR-223 include IGF1R, Iκκ α, NLPR3, Pknox1, Roquin, STAT3, etc (Fukao et al., 2007). The downstream cell signal transduction pathway, JAK/STAT, is of great importance in the normal physiological processes and pathological processes of vascular diseases. The current research is aimed on the role of STAT3 pathway in the occurrence and development of AS (Cho et al., 2015; Qin et al., 2015; Dodington et al., 2018). A variety of activated factors, such as IL-5, IL-6, IL-10, interferon, can activate STAT3 pathway and regulate cell proliferation, migration, infiltration, adhesion, invasion, differentiation, apoptosis, inflammation, and related biological activity (Chen et al., 2012; Kesanakurti et al., 2013; Johnson et al., 2018). Our study confirm the negative regulation of miR-223 on STAT3 pathway in HUVECs. Data displayed that paeonol could inhibit STAT3 pathway in both exosomes and HUVECs, which could be inverted after miR-223 inhibitor transfection into THP-1 cells.

Our study suggested exosomes and its cargo microRNA as a new target for treatment of AS. Nevertheless, it will be a challenge to determine how to induce moderate exosomal miR-223 expression in the human body, which will require more research.

## Conclusion

To sum up, our current results showed that paeonol could increase the monocytes-derived exosomal miR-223 in HUVECs, thus ultimately down-regulate the secretion of IL-1β, IL-6, VCAM-1, ICAM-1 in HUVECs and the adhesion function between monocyte and HUVECs via inhibiting STAT3 pathway in HUVECs. So far we verified the role of monocytes-derived exosomal miR-223 in inflammatory response of VECs and the underlying mechanisms of paeonol’s protective effect on AS.

## Author Contributions

YL wrote the paper. YL and CL performed the *in vitro* experiments. HW and XX performed the *in vivo* experiments. YL and YS prepared the figures. YL and CL analyzed and commented the results. MD designed the study and supervised the project. All the authors have read and approved the final manuscript.

## Conflict of Interest Statement

The authors declare that the research was conducted in the absence of any commercial or financial relationships that could be construed as a potential conflict of interest.
