# Supplementary figures and images for: Paeonol Attenuated Inflammatory Response of Endothelial Cells via Stimulating Monocytes-Derived Exosomal MicroRNA-223
Source: Front Pharmacol. 2018 Nov 20;9:1105. doi: 10.3389/fphar.2018.01105 (PMC6256086; doi:10.3389/fphar.2018.01105)

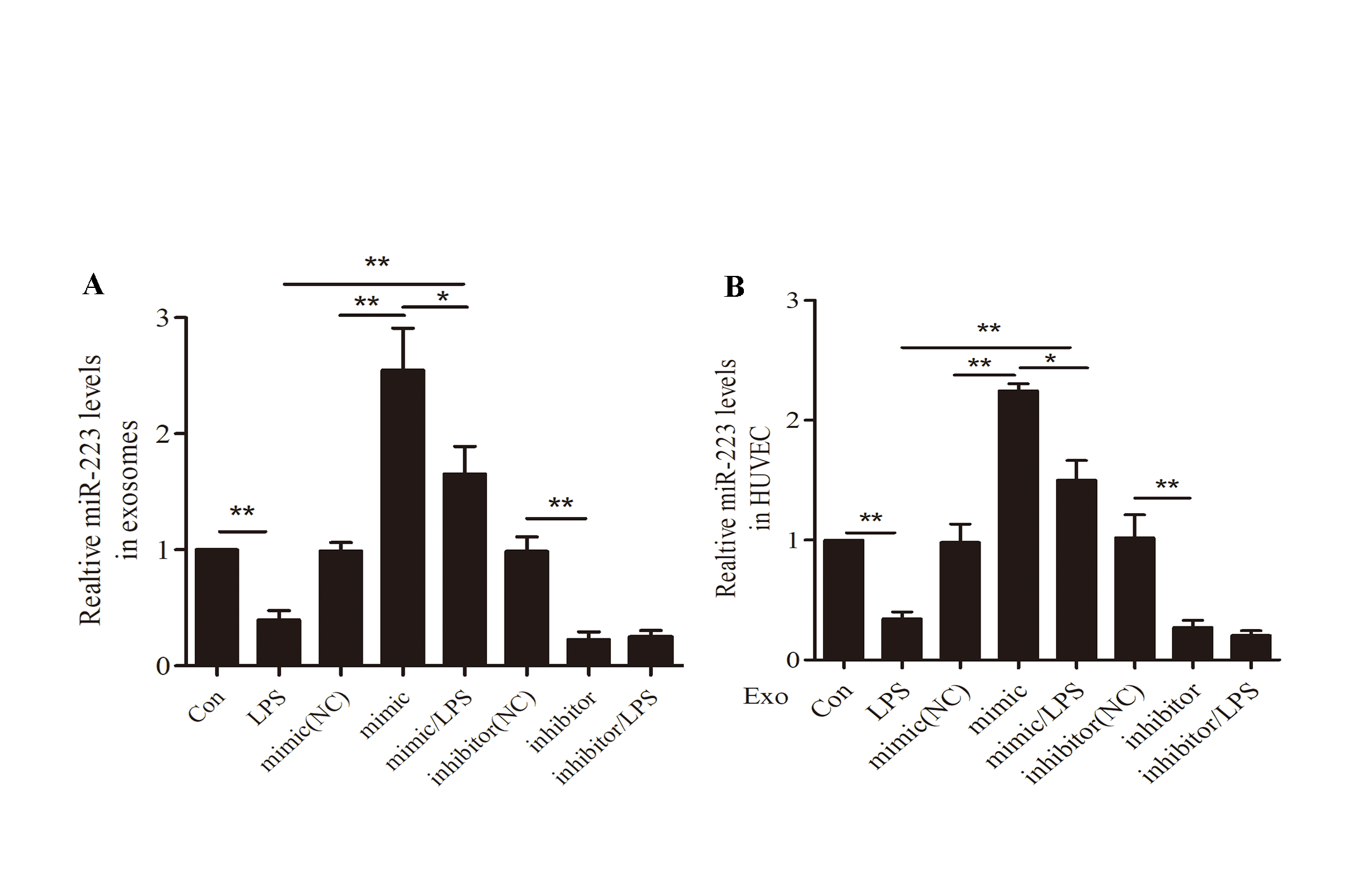

Supplement: Supplementary file 1 [file Image_1.TIF]

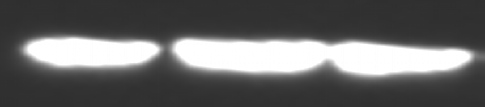

Supplement: Supplementary file 3 [file Presentation_1.ZIP › original Western blots images/Fig. 1C/B-actin.tif]

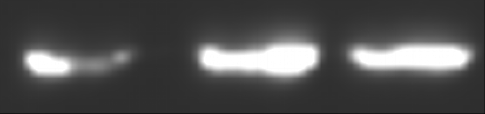

Supplement: Supplementary file 3 [file Presentation_1.ZIP › original Western blots images/Fig. 1C/ICAM-1.tif]

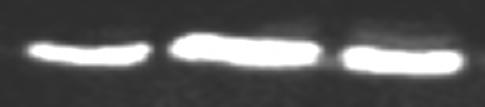

Supplement: Supplementary file 3 [file Presentation_1.ZIP › original Western blots images/Fig. 1C/VCAM-1.tif]

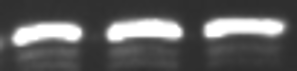

Supplement: Supplementary file 3 [file Presentation_1.ZIP › original Western blots images/Fig. 1D/B-actin.tif]

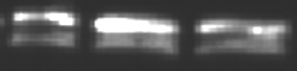

Supplement: Supplementary file 3 [file Presentation_1.ZIP › original Western blots images/Fig. 1D/p-STAT3.tif]

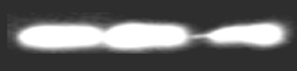

Supplement: Supplementary file 3 [file Presentation_1.ZIP › original Western blots images/Fig. 1D/STAT3.tif]

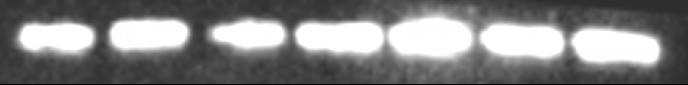

Supplement: Supplementary file 3 [file Presentation_1.ZIP › original Western blots images/Fig. 2C/B-actin.tif]

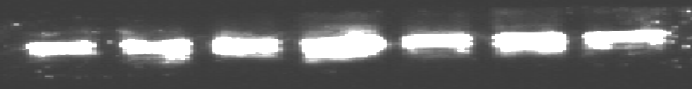

Supplement: Supplementary file 3 [file Presentation_1.ZIP › original Western blots images/Fig. 2C/ICAM-1.tif]

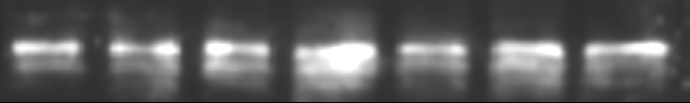

Supplement: Supplementary file 3 [file Presentation_1.ZIP › original Western blots images/Fig. 2C/VCAM-1.tif]

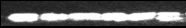

Supplement: Supplementary file 3 [file Presentation_1.ZIP › original Western blots images/Fig. 2F/B-actin.tif]

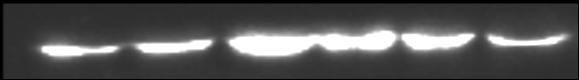

Supplement: Supplementary file 3 [file Presentation_1.ZIP › original Western blots images/Fig. 2F/ICAM-1.tif]

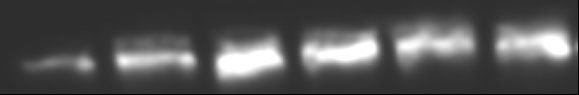

Supplement: Supplementary file 3 [file Presentation_1.ZIP › original Western blots images/Fig. 2F/VCAM-1.tif]

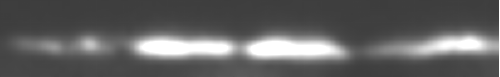

Supplement: Supplementary file 3 [file Presentation_1.ZIP › original Western blots images/Fig. 4/Alix.tif]

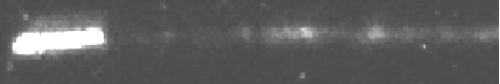

Supplement: Supplementary file 3 [file Presentation_1.ZIP › original Western blots images/Fig. 4/Calnexin.tif]

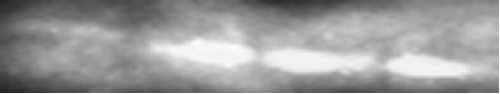

Supplement: Supplementary file 3 [file Presentation_1.ZIP › original Western blots images/Fig. 4/CD63.tif]

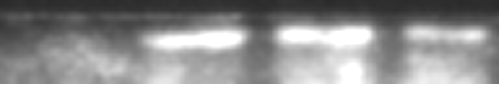

Supplement: Supplementary file 3 [file Presentation_1.ZIP › original Western blots images/Fig. 4/CD9.tif]

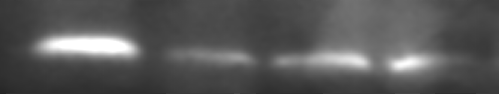

Supplement: Supplementary file 3 [file Presentation_1.ZIP › original Western blots images/Fig. 4/HSP70.tif]

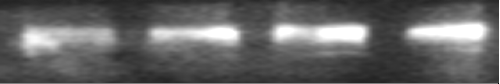

Supplement: Supplementary file 3 [file Presentation_1.ZIP › original Western blots images/Fig. 4/Lamp2.tif]

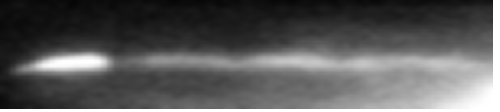

Supplement: Supplementary file 3 [file Presentation_1.ZIP › original Western blots images/Fig. 4/TSG101.tif]

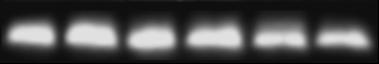

Supplement: Supplementary file 3 [file Presentation_1.ZIP › original Western blots images/Fig. 5F/B-actin.tif]

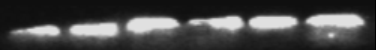

Supplement: Supplementary file 3 [file Presentation_1.ZIP › original Western blots images/Fig. 5F/ICAM-1.tif]

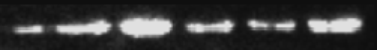

Supplement: Supplementary file 3 [file Presentation_1.ZIP › original Western blots images/Fig. 5F/VCAM-1.tif]

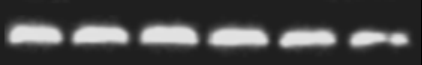

Supplement: Supplementary file 3 [file Presentation_1.ZIP › original Western blots images/Fig. 5G/B-actin.tif]

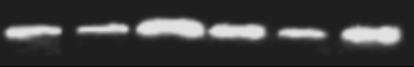

Supplement: Supplementary file 3 [file Presentation_1.ZIP › original Western blots images/Fig. 5G/p-STAT3.tif]

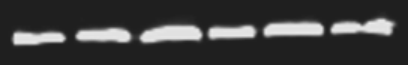

Supplement: Supplementary file 3 [file Presentation_1.ZIP › original Western blots images/Fig. 5G/STAT3.tif]

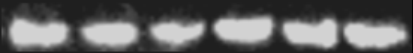

Supplement: Supplementary file 3 [file Presentation_1.ZIP › original Western blots images/Fig. 6/B-actin.tif]

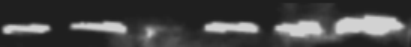

Supplement: Supplementary file 3 [file Presentation_1.ZIP › original Western blots images/Fig. 6/p-STAT3.tif]

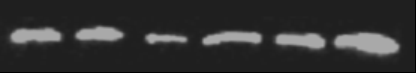

Supplement: Supplementary file 3 [file Presentation_1.ZIP › original Western blots images/Fig. 6/STAT3.tif]

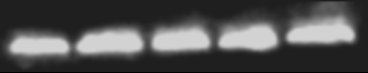

Supplement: Supplementary file 3 [file Presentation_1.ZIP › original Western blots images/Fig. 7D/B-actin.tif]

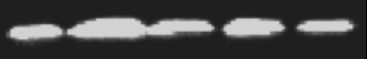

Supplement: Supplementary file 3 [file Presentation_1.ZIP › original Western blots images/Fig. 7D/ICAM-1.tif]

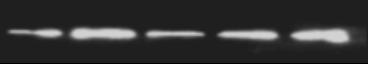

Supplement: Supplementary file 3 [file Presentation_1.ZIP › original Western blots images/Fig. 7D/VCAM-1.tif]

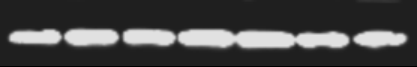

Supplement: Supplementary file 3 [file Presentation_1.ZIP › original Western blots images/Fig. 7E/B-actin.tif]

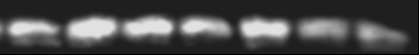

Supplement: Supplementary file 3 [file Presentation_1.ZIP › original Western blots images/Fig. 7E/p-STAT3.tif]

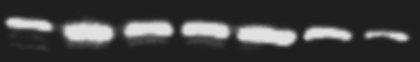

Supplement: Supplementary file 3 [file Presentation_1.ZIP › original Western blots images/Fig. 7E/STAT3.tif]
