# Supplementary material for: Paeonol Attenuated Inflammatory Response of Endothelial Cells via Stimulating Monocytes-Derived Exosomal MicroRNA-223
Source: Front Pharmacol. 2018 Nov 20;9:1105. doi: 10.3389/fphar.2018.01105 (PMC6256086; doi:10.3389/fphar.2018.01105)
Supplement: Supplementary file 2 [file Table_1.DOC]

**Figure**. Effects of cell transfection on miR-223 expression in exosomes and HUVECs. The data were expressed as mean±SEM, n=3. We performed transfection of miR-223 mimic or inhibitor to THP-1 cells, then THP-1 cells were treated by LPS (1 µg/mL) for 24 h. Exosomes were extracted from THP-1 cells to establish the co-culture system of exosomes-HUVECs. **(A)** miR-223 levels in exosomes. **(B)** miR-223 levels in exosomes in HUVECs. ^*^*P*<0.05, ^**^*P*<0.01 vs. Con group.
